# Optimization and Analysis of Centrifugal Pump considering Fluid-Structure Interaction

**DOI:** 10.1155/2014/131802

**Published:** 2014-08-13

**Authors:** Yu Zhang, Sanbao Hu, Yunqing Zhang, Liping Chen

**Affiliations:** ^1^Wuhan Second Ship Design and Research Institute, Wuhan, Hubei 430064, China; ^2^Hubei Key Laboratory of Advanced Technology of Automobile Parts, Wuhan University of Technology, Wuhan, Hubei 430074, China; ^3^Center for Computer-Aided Design, School of Mechanical Science & Engineering, Huazhong University of Science & Technology, Wuhan, Hubei 430074, China

## Abstract

This paper presents the optimization of vibrations of centrifugal pump considering fluid-structure interaction (FSI). A set of centrifugal pumps with various blade shapes were studied using FSI method, in order to investigate the transient vibration performance. The Kriging model, based on the results of the FSI simulations, was established to approximate the relationship between the geometrical parameters of pump impeller and the root mean square (RMS) values of the displacement response at the pump bearing block. Hence, multi-island genetic algorithm (MIGA) has been implemented to minimize the RMS value of the impeller displacement. A prototype of centrifugal pump has been manufactured and an experimental validation of the optimization results has been carried out. The comparison among results of Kriging surrogate model, FSI simulation, and experimental test showed a good consistency of the three approaches. Finally, the transient mechanical behavior of pump impeller has been investigated using FSI method based on the optimized geometry parameters of pump impeller.

## 1. Introduction

Centrifugal pumps provide the energy to move fluids through piping systems, including equipment, piping, and fittings and through elevation changes in open systems. Centrifugal pumps have been widely used in various industrial applications, such as oil and gas, agriculture, chemistry, and marine industry as well as metallurgy. Because of the customers' increasing demands of high-quality pump, optimization design of centrifugal pump plays an important role in pump industry, and there have been many efforts to optimize the performance of centrifugal pump in recent years. Anagnostopoulos [1] proposed an optimization algorithm based on unconstrained gradient method to find the impeller geometry that could maximize the pump efficiency among a set of blade angles. Zhou et al. [2] optimized the geometric shape of the centrifugal impeller using orthogonal experiment method to improve the performance of the centrifugal pump. Derakhshan et al. [3] presented the incomplete sensitivities approach and genetic algorithms to obtain a higher efficiency by redesigning the shape of impeller blades. Papierski and Blaszczyk [4] decomposed the optimization design of centrifugal pump into two levels to maximize the efficiency and simultaneously minimize required net positive suction head (NPSHr). These researches mainly focus on optimizing the performance data of centrifugal pump, such as head, efficiency, or NPSHr. However, the vibration performance is important especially for high-pressure centrifugal pump.

The vibration that occurs while centrifugal pump works can cause fatigue and damage of pump components and weaken the operation stability. Vibrations of centrifugal pump have attracted interest of researchers. For example Hodkiewicz and Norton [5] investigated the influence of different flow rates on the vibration performance of double-suction centrifugal pump. Guo and Maruta [6] presented an experimental study of the pressure fluctuation and the impeller vibration in a centrifugal pump with some vaned diffusers. Rodriguez et al. [7] developed a theoretical analysis approach to investigate the vibrating frequencies in the vibration of centrifugal pump induced by the rotor stator interaction (RSI). Wang et al. [8] studied the structural dynamics characteristics and vibrations of pump volute casing for a double-suction centrifugal pump using a fluid-structure coupling interface model.

Studies agree in considering the fluid-structure interaction (FSI) as the source of the highest vibration levels in large centrifugal pumps. Moreover, hydraulic excitation forces are due to the FSI and cause pressure fluctuations, mechanical vibrations, and alternating stresses in different components of centrifugal pump. In recent years, the application of FSI theory to centrifugal pumps became more popular and it is well documented in literature [9–13].

Vibration performance is one of the most important parameters in designing a centrifugal pump. Actually, experimental tests and CFD simulation are the two methods performed in order to obtain the centrifugal pump vibration response. However, both of the two methods cannot be considered in optimizing the vibration performance of the pump using an iterative method. Appropriate metamodels must be established between the decision variables and the concerned objective functions. Therefore, metamodel technique demonstrates its superiority in the optimization problem of engineering.

Kriging metamodels [14] were originally proposed by the South African mining engineer named Danie Gerhardus Krige. With the rapid development of computer technology, Kriging metamodels have been widely used in various fields [15–19]. Kriging metamodel differs from other metamodels because of the optimal unbiased prediction for the unknown response points [20]. Compared to traditionally response surface methods, Kriging shows its superiority in high dimensional nonlinear problems and prediction accuracy due to the stochastic assumption [21], especially for multiobjective optimization problems [22].

This paper presents an effective optimization method based on Kriging metamodel. The presented method optimizes the vibration performance of the centrifugal pump undergoing FSI phenomena, which reasonably take advantages of the FSI simulation, Kriging metamodel and experimental tests. Although considerable researches were devoted to investigating the vibration performance of centrifugal pump, it should be noted that there exists little literature evidence on the vibration optimization of centrifugal pump in particular that combines with FSI phenomenon. The second part of the paper deals with the study of the transient mechanical characteristics based on the optimized centrifugal pump using FSI method.

## 2. Centrifugal Pump FSI Simulation Model

### 2.1. FSI Governing Equations

In this study, the fluid-structure interaction (FSI) problem's domain *Ω* consists of two subdomains: *Ω*_*f*_ and *Ω*_*s*_ with the boundaries as Γ_*f*_ and Γ_*s*_, respectively. The subscripts *f* and *s* denote the fluid part and solid part, respectively. The following section defines the equations that govern the flow and the structural deformations of the pump.

#### 2.1.1. Fluid Flow Equations

The fluid flowing through the centrifugal pump is treated as incompressible and isothermal. For **x** ∈ *Ω*_*f*_, the conservation of mass and Navier-Stokes equations governing the unsteady flow are, respectively, written as

(1) $$\begin{array}{c} \frac{\partial \rho_{f}}{\partial t}+\nabla \cdot \left(\rho_{f}v_{f}\right)=0,\\ \rho_{f}\frac{\partial v_{f}}{\partial t}+\rho_{f}\left(v_{f}\cdot \nabla \right)v_{f}-\nabla \cdot \sigma_{f}=f_{f}, \end{array}$$

where *ρ*_*f*_ is the fluid density, **v**_*f*_ denotes the fluid particle velocity at time *t*, **f**_*f*_ denotes the body forces per unit of volume on the fluid, and **σ**_*f*_ is the stress tensor defined as

(2) $$\begin{array}{c} \sigma_{f}=-pI+\mu \left[\nabla v_{f}+{\left(\nabla v_{f}\right)}^{T}\right], \end{array}$$

where *p* is the pressure, **I** denotes the unit tensor, and *μ* represents the absolute viscosity.

#### 2.1.2. Structural Equations

While working, the centrifugal pump undergoes large deformation and rotation. For **x** ∈ *Ω*_*s*_, the conservation of momentum for the solid deformation **u**_*s*_ is described through Lagrangian formulation:

(3) $$\begin{array}{c} \rho_{s}\frac{\partial^{2}u_{s}}{\partial t^{2}}=\nabla \cdot \sigma_{s}+f_{s}, \end{array}$$

where *ρ*_*s*_ is the solid density, **σ**_*s*_ represents the Cauchy stress tensor, and **f**_*s*_ denotes the body forces per unit volume on the solid.

Closure for (3) is found by evaluating the stress using the relevant constitutive relations. Moreover, since the centrifugal pump is related to large deformation and rotation, the constitutive equations are described using a stress-strain relationship.

#### 2.1.3. Interaction between Fluid and Solid

As mentioned above, the FSI occurs during the running process of centrifugal pump. Fluid pressure information transfers to the solid, while displacements information of the solid transfers to the flow. Furthermore, on the no-slip fluid-structural interface, the information exchange between the fluid and solid should follow the equilibrium conditions

(4) $$\begin{array}{c} \begin{array}{c} \;v=\frac{\partial u}{\partial t}\\ \;\sigma_{f}\cdot n=\sigma_{s}\cdot n \end{array}\;\forall x\in \Gamma , \end{array}$$

where Γ denotes the fluid-structural interface and Γ = Γ_*f*_∩Γ_*s*_, **n** represents the unit normal at the interface Γ.

### 2.2. Decision Variables

The working process of centrifugal pump involves vibrations, FSI, and energy conversion and loss. As the “heart” component for a centrifugal pump, impeller plays an important role in all these phenomena and transforms the mechanical energy into the kinetic energy of the fluid. Moreover, the geometry shape of impeller blade has strong effect on pump performance, including vibrations. This paper focuses on optimizing the impeller blade to minimize the vibration response of the centrifugal pump.

The recommended number of impeller blades for high head centrifugal pumps is usually between five and seven. In fact, too many blades lead to higher friction losses and may cause low blade loading; fewer blades may result in higher blade loading. Turbulent dissipation losses will rise because of the increased secondary flow and stronger deviation between blade and flow direction. Therefore, six blades are chosen.

**Figure 1:**
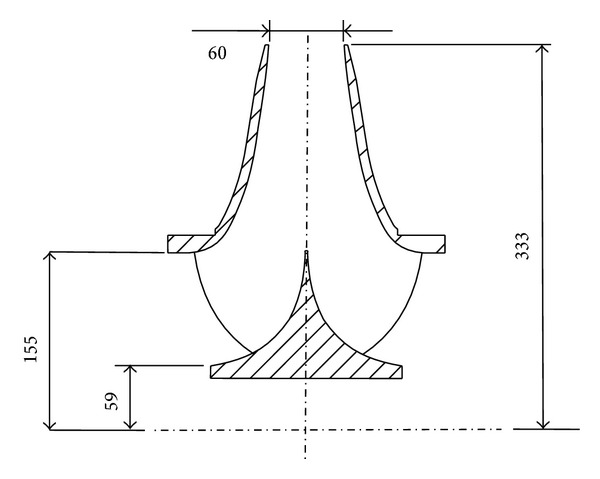
Main dimensions of centrifugal pump's impellers (unit: mm).

Figure 1 shows the main dimensional parameters of the impellers of the centrifugal pump studied in this paper, notably, it is double suction type. In addition, among all kinds of centrifugal pumps, the double suction centrifugal pumps are widely used in industry for various applications due to large flux and high lift.

**Figure 2:**
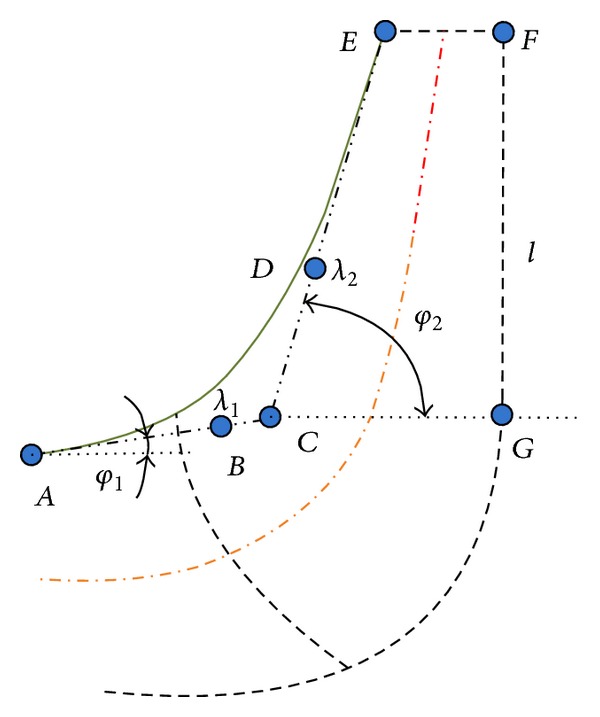
Meridional section of the pump impeller.

Figure 2 shows the meridional section of the impeller. Due to symmetry of the model, the optimization of double suction impeller can be converted into the optimization of a single suction type. Hence, as Figure 2 shows, the meridional section of the single suction impeller is actually determined by the solid line and dashed line $\bar{FG}$. The solid line is parameterized by quartic Bézier curve with five control points; the five decision variables for the pump impeller are *φ*_1_, *λ*_1_, *φ*_2_, *λ*_2_, and *l*.  Table 1 summarizes the boundaries of the decision variables. Here, *λ* is the relative position in the line segment. For example, taking line segment $\bar{AC}$, *λ*_1_ is the ratio of the line length $\bar{AB}$ to $\bar{AC}$. *l* is the length of line segment $\bar{FG}$.

### 2.3. FSI Model Sample and Simulation

Latin hypercube sampling (LHS) is a design of experiment (DOE) method originally developed by Mckay in 1979. LHS approach has the space-filling character and can guarantee the sample points covering the entire design domains homogeneously. Hence, 119 simple points and 30 test points have been obtain by LHS method. The simple points are the input data of Kriging surrogate model, while the test points are used to validate the accuracy of the Kriging predictor.

**Figure 3:**
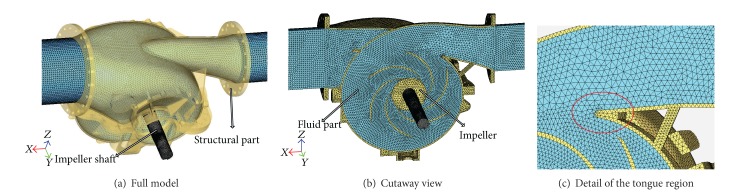
One case of FSI simulation models.

Table 2 summarizes the combinations of decision variables in the sample points. The FSI simulation models are built based on these sample points.  Figure 3 shows one case of FSI simulation models.  Figure 3(a) corresponds to a full FSI model with solid and fluid parts.  Figure 3(b) is the cutaway view of the full FSI model, and Figure 3(c) gives the detailed view of the tongue region. The structural part consists of pump volute casing, impeller, and impeller shaft, while the fluid part is the liquid flowing through the structural part. Moreover, the fluid part is also called the hydraulic model of centrifugal pump.

The calculation of structural part of the pump has been carried out through computational structure dynamics (CSD) analysis, performed using Abaqus FEA software. The pump volute casing and impeller are both made of aluminum-bronze alloy; the elastic modulus is 125000 MPa, the density is 7630 Kg/m^3^, and the Poisson's ratio is 0.327. The impeller shaft is made of alloy steel, with elastic modulus of 206000 MPa, density of 7800 Kg/m^3^, and Poisson's ratio of 0.3. The increment size of time step is set as 1 × 10^−4^ s, and the total simulation time is 6*T*, where *T* is the cycle of the pump corresponding to a changed angle of 60°. Furthermore, the differential equation of the centrifugal pump at excitation state by FSI can be expressed as

(5) $$\begin{array}{c} M\ddot{x}\left(t\right)+C\dot{x}\left(t\right)+Kx\left(t\right)=F\left(t\right), \end{array}$$

where *t* is the time; **M**, **C**, and **K** are the structural mass matrix, structural damping matrix and stiffness matrix, respectively; $\ddot{x}\left(t\right)$, $\dot{x}\left(t\right)$, and **x**(*t*) represent the acceleration vector, velocity vector, and displacement vector, respectively; **F**(*t*) denotes the load vector of the node.

The computational fluid dynamics (CFD) has been simulated using Fluent code. The fluid is water, with a temperature of 20°C, density of 998.2 Kg/m^3^, and viscosity of 1.003 × 10^−3^ Pa*·*s.  Table 3 lists the parameters for CFD simulations. The hydraulic models are established by the standard *k* − *ε* turbulence models and wall functions based on logarithmic law, which are consistent with the no-slip condition. Static boundary condition and rotary boundary condition are imposed on the boundary of volute flow domain and impeller flow domain, respectively. Moreover, the interaction between these two boundaries is taken into account through the moving mesh model. The unsteady Reynolds-averaged Navier-Stokes (URANS) equations are calculated by finite volume method (FVM), and the pressure-velocity coupling is solved by means of the SIMPLEC algorithm. Second order upwind discretizations are used for determine and diffusive terms of the turbulence model equations. The residual error is set as 1 × 10^−5^ to judge whether the calculation is convergent. In addition, the time step and total simulation time are set as 1 × 10^−4^ s and 6*T*, respectively, in order to correspond with the structural simulation part.

**Figure 4:**
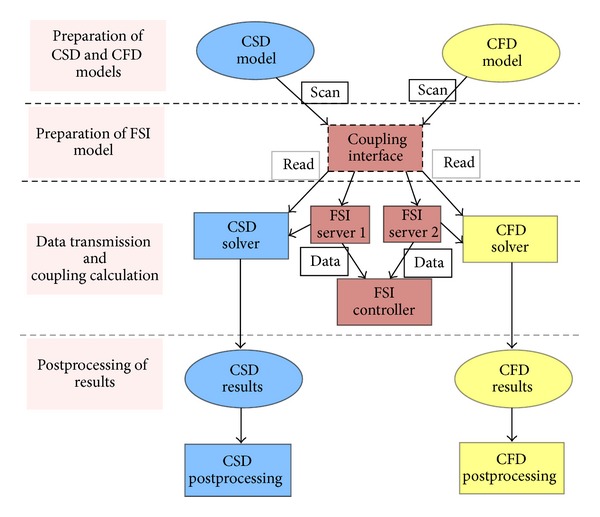
The process of FSI simulation.

The information exchange between solid (Abaqus) and fluid flow (Fluent) at the coupling interface is performed in the platform of MpCCI.  Figure 4 outlines the process of FSI simulation: first, the models of CSD and CFD are prepared independently, such as the setting of material, loads, and boundary conditions. Then, the pressure information of the fluid is transferred to Abaqus for structure analysis; meanwhile, the displacements information of the structure are transferred to Fluent for fluid analysis, and the information exchange at the coupling interface repeatedly until the calculation has converged. Finally, the results of FSI simulation are post processed using both Abaqus and Fluent.

As aforementioned, this paper mainly focuses on optimizing the vibration performance of the centrifugal pump using FSI. Hence, the root mean square (RMS) value of the displacement response at the pump bearing block is chosen as the objective function, which can be defined as follows:

(6) $$\begin{array}{c} U_{\text{RMS}}=\sqrt{\frac{1}{N}\overset{N}{\underset{i=1}{\sum }}U_{i}^{2}}, \end{array}$$

where *N* is the total number of the time steps, and *U*_*i*_ denotes the displacement response of the *i*th time step, and the direction is the vertical direction of the bearing support. The last column of Table 2 summarizes the results of *U*_RMS_ calculated through FSI simulations at the sample points, which are the output data used to build the Kriging model.

## 3. Kriging-Based Optimization

### 3.1. Kriging Approach

Kriging predicts unknown values of a random function based on all of the observed points [22]. Moreover, Kriging metamodels show global performance rather than local characteristics. A combination of a global model and localized departures of the form describes a Kriging model; thus,

(7) $$\begin{array}{c} y\left(x\right)=\overset{n}{\underset{k=1}{\sum }}\beta_{k}f_{k}\left(x\right)+Z\left(x\right), \end{array}$$

where *y*(*x*) is the response function, **f**(*x*) = [*f*_1_(*x*),…,*f*_*n*_(*x*)]^*T*^ is the regression basis function, *n* is the number of the basis function, and **β** = [*β*_1_,…,*β*_*n*_]^*T*^ is the regression coefficient. *Z*(*x*) is assumed as a realization of an independent Gaussian random process with zero mean and spatial correlation function given by [23]

(8) $$\begin{array}{c} \text{Co}\;v\left[Z\left(\tau \right),Z\left(x\right)\right]=\sigma^{2}R\left(\theta ,\tau ,x\right), \end{array}$$

where *σ* ^2^ denotes the process variance, *R*(*θ*, *τ*, *x*) is the correlation function between the points *τ* and *x*, and *θ* is the unknown correlation parameter. Several types of correlation models, such as linear correlation model and exponential correlation model can be considered. However, the Gauss correlation model adopted in this paper is more popular in Kriging metamodels with the form

(9) $$\begin{array}{c} R\left(\theta ,\tau ,x\right)=\exp\left(-\overset{m}{\underset{j=1}{\sum }}\theta_{j}{\left(\tau_{j}-x_{j}\right)}^{2}\right), \end{array}$$

where the quantities *τ*_*j*_ and *x*_*j*_, respectively, denote the *j*th components of sample points *τ* and *x*; *m* is the dimension of the decision variables.

The predicted value and estimation error at point *x* are, respectively, given by

(10) $$\begin{array}{c} \hat{y}\left(x\right)=f^{T}\left(x\right)\hat{\beta }+r^{T}\left(x\right)R^{-1}\left(Y-F\hat{\beta }\right),\\ s\left(x\right)=\sigma^{2}\left(1+u^{T}{\left(F^{T}R^{-1}F\right)}^{-1}u-r{\left(x\right)}^{T}R^{-1}r\left(x\right)\right), \end{array}$$

where **Y** represents the response of the sample points, **u** = **F** ^*T*^ **R** ^−1^ **r**(*x*) − **f**(*x*), **F** is a vector which is composed by the value of **f**(*x*) at each sample point, **r** ^*T*^(*x*) denotes a vector which represents the correlation between an unknown point and all known sample points. In addition, **r** ^*T*^(*x*) = [*R*(*θ*, *x*, *x*_1_) ⋯ *R*(*θ*, *x*, *x*_*N*_)], *N* is the total number of the sample points, **R** is an *N* × *N* symmetric correlation matrix written in the following form:

(11) $$\begin{array}{c} R=\left[\begin{array}{ccc} R\left(x_{1},x_{1}\right) & \cdots & R\left(x_{1},x_{N}\right)\\ \vdots & \ddots & \vdots \\ R\left(x_{N},x_{1}\right) & \cdots & R\left(x_{N},x_{N}\right) \end{array}\right]. \end{array}$$

Under the unbiased condition, the unknown parameters **β** and *σ* ^2^ can be estimated through

(12) $$\begin{array}{c} \hat{\beta }={\left(F^{T}R^{-1}F\right)}^{-1}F^{T}R^{-1}Y,\\ {\hat{\sigma }}^{2}=\frac{1}{m}{\left(Y-F\hat{\beta }\right)}^{T}R^{-1}\left(Y-F\hat{\beta }\right). \end{array}$$

As a matter of fact, once the types of regression model and correlation model have been chosen, the correlation matrix **R** and unknown parameters **β** and *σ* ^2^ all depend on the correlation parameter *θ*. Thus, a Kriging metamodel is completely established only if the value of *θ* is determined. Furthermore, the most commonly used approach to calculate the value of correlation parameter *θ* is maximum likelihood estimation (MLE), and the problem can be converted into an unconstrained global optimization problem as follows [20]:

(13)  Minimize {ψ(θ)=σ(θ)2|Rθ|1/m} Subject  to θ>0.

### 3.2. Modeling and Verification of the Kriging Model

Kriging metamodel is established according to Table 2. The input data is the set of 119 sample points obtained through LHS method, and the input variables are the impeller geometric parameters *φ*_1_, *λ*_1_, *φ*_2_, *λ*_2_, and *l* shown in Figure 2. The output data are the results of FSI simulations corresponding to the sample points, and the output variable is the RMS value of the displacement response *U*_RMS_.  Table 4 shows the parameters of the Kriging metamodel, **β**, *σ* ^2^, and ***θ***.

The Kriging metamodel can be applied to the vibration optimization only if the Kriging predictor's estimated accuracy is higher enough. Otherwise, the metamodel should be rebuilt by adjusting the parameters. An additional set of 30 points obtained through LHS method is used as test points to verify the performance of Kriging's predictor. The FSI analysis gives the RMS values of the displacement response corresponding with the test points. In addition, for the FSI simulations based on the test points, the basic parameters and boundary conditions are the same with the sample points.

**Figure 5:**
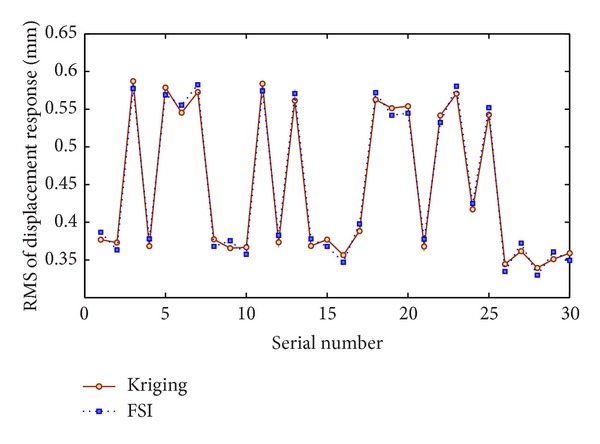
The results of RMS values at test points.

Figure 5 shows the results of the displacement response's RMS values obtained by Kriging predictor and FSI simulations at the test points. Results show that the predicted values of the Kriging metamodel correspond to the FSI simulation values. Hence, the vibration optimization of centrifugal pump can be performed based on the Kriging surrogate model.

### 3.3. Optimization Based on the Surrogate Model

The optimization problem of centrifugal pump in this paper can be given as follows:

(14) Find    X=[φ1,λ1,φ2,λ2,l]TMinimize  URMS=f(φ1,λ1,φ2,λ2,l)Subject  to  0°≤φ1≤30°0.02≤λ1≤0.9870°≤φ2≤90°0.02≤λ2≤0.98145 mm≤l≤195 mm,

where is *f* the Kriging approximation of the displacement response's RMS values.

The above-defined problem can be resolved through multi-island genetic algorithm (MIGA), a modified version of genetic algorithm (GA). MIGA decomposes the population in one generation into several subpopulations. The subpopulations are also called “Islands,” and the genetic operations are executed on each “Island” independently. Furthermore, this independency can prevent the optimization solution from local optima.  Table 5 lists the detailed parameter settings used for MIGA. The experiments are carried out on a Desktop PC with Intel Core 2 quad CPU and 3.25 GB RAM; all of the cores have the speed of 2.66 GHz. Due to stochastic behavior of MIGA algorithm, at least 30 independent runs are required to provide the results with statistical confidence.

## 4. Result and Discussion

### 4.1. Optimization Result and Validation

**Figure 6:**
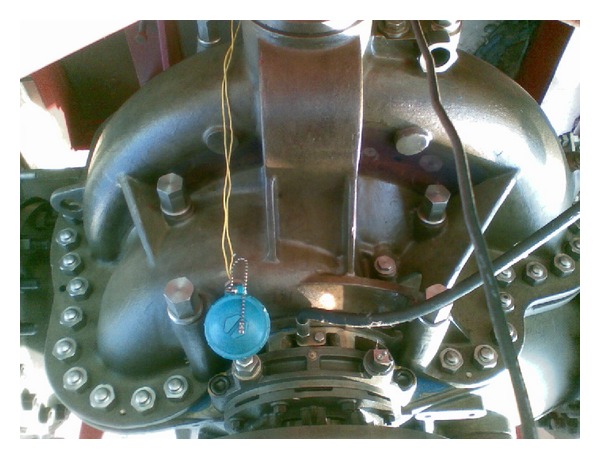
The prototype of centrifugal pump corresponding to the optimization result.

Table 6 shows the optimization result and average CPU time. The RMS value of the displacement response improves to 0.3341 mm. Moreover, the accuracies of Kriging metamodel and FSI simulation have been further validated through experimental tests. Thus, a prototype of centrifugal pump based on the geometric parameters in Table 6 has been produced, as shown in Figure 6. The pump is fixed on the test bench; a 1 MW electric machinery drives the pump impeller. A displacement sensor installed at the pump bearing measures the displacement response of the bearing block. The water circulates in a close loop and the flow rate is constant. The basic test parameters correspond to parameters of FSI simulations summarized in Table 3.

Table 7 compares the results of Kriging predictor, FSI simulation, and experimental test. The results given by the three methods well agree to each other. The error of Kriging metamodel is 3.1%, the error of FSI simulation is 4.4%, and they are both less than 5%. It is well known that the experimental test plays an indispensable role in validating the optimization design for centrifugal pump. However, manufacturing a prototype pump or the experimental equipment is expensive. Furthermore, due to the complexity of the model, the FSI simulation is time-consuming. Hence, the optimized design of the pump should minimize both elements: costs and calculation time.

This research shows that the predictive ability of the Kriging model has been well justified both by FSI simulations and by experimental test. Therefore, the well validated surrogate model can completely replace time-consuming FSI simulations and substitute a great majority of expensive experiment tests. That is, the Kriging surrogate model provides great convenience in studying the vibration performance of centrifugal pump, especially for accumulating the practical experience of pump design. Moreover, the well validated surrogate model can benefit both the further development of centrifugal pump manufacturer and the improvement of the pump designer's ability. Therefore, the surrogate model method makes the investigation of pump performance easy, which is of course on the promise that the model accuracy is high enough.

### 4.2. The Analysis of Impeller Mechanical Behavior through FSI

The mechanical characteristics of the pump impeller are significant for the working behaviors of centrifugal pumps. During the working process of a centrifugal pump, the periodic hydraulic loads imposed on the pump will lead to the dynamic deformation of the impeller and impeller shaft. Moreover, the dynamic deformation will further influence the flow field distribution. The analysis of the mechanical behavior of the impeller is a typical FSI problem. In general, there are mainly three types of loads acting on pump impeller: coupling pressure load from the fluid, gravity, and inertia force due to the circular motion. However, all these loads are finally balanced by the support reaction of the bearings and the input moment of the pump. FSI method allows investigating the dynamic force of the impeller and the input moment, and the calculation results are important to highlight the mechanical properties of the centrifugal pump. For example, the analysis results can help in choosing the appropriate sizes and types of impeller shaft and bearings.

Actually, either the radial force of the pump impeller or the input moment of the pump cannot be easily measured because of the expensive measuring equipment and complex multipoints installation. When the simulation model is accurate, FSI simulation method shows advantage in obtaining the radial force and input moment. Furthermore, as previously mentioned in Section 4.1, the results obtained by Kriging predictor's, FSI simulation, and experiment test well agree to each other. The comparison indicates that the FSI simulation model is well validated, and FSI simulation model leads to reliable results. This research investigates the radial force of the pump impeller and the input moment of the pump through FSI method. In addition, the radial force and input moment are calculated based on the FSI simulation model in Section 4.1. The basic settings of the FSI simulation are unchanged, such as the definitions of material properties, simulation time step, boundary condition, and coupling interface.

**Figure 7:**
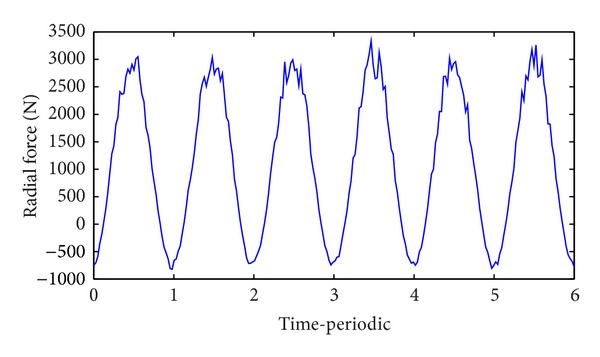
The radial force of the pump impeller.

**Figure 8:**
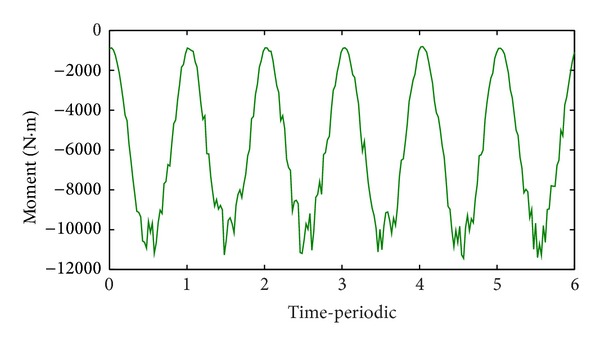
The input moment of the pump.

Figures 7 and 8 show the results of dynamic radial force of pump impeller and dynamic input moment of the pump, respectively. The time-dependent transient force and moment in each time step are calculated by direct integration method. Both the curve of radial force and moment indicate cyclical fluctuation in general and six cycles corresponding to a full pump impeller revolution. However, as a result of the tongue region shown in Figure 3(c), there exists more or less disturbance on the wave crest or wave trough.

## 5. Conclusions

This paper proposes a Kriging-based optimization method for the vibrations optimization of centrifugal pumps, which well integrates Kriging surrogate model, FSI simulations, and experimental tests. Moreover, the proposed method overcomes the faults of expensive computation and cost, and it has been proved to be effective on improving pump vibration performance in terms of minimum cost and reduction of development period.

The Kriging surrogate model of pump vibration performance has been established based on the sample points, and the results at the test points showed that the Kriging predictor well agreed with the FSI simulations. The final optimized decision variables have been obtained using MIGA; a prototype has been manufactured according to optimized values of geometrical parameters of the pump. Experimental tests carried out on prototype well agreed with the results of Kriging metamodel and FSI simulation.

Furthermore, based on the final optimized decision variables, the dynamic mechanical performance of pump impeller was further investigated using FSI method. The results showed that the radial force curve and moment curve exhibited cyclical fluctuation.

## Figures and Tables

**Table 1:** Decision variables and their boundaries.

Decision variable	Lower boundary	Upper boundary
*φ*_1_ (deg)	0	30
*λ*_1_	0.02	0.98
*φ*_2_ (deg)	70	90
*λ*_2_	0.02	0.98
*l* (mm)	145	195

**Table 2:** The sample points and corresponding results of FSI simulations.

Serial number	Decision variable					Objective
	*φ*_1_ (deg)	*λ*_1_	*φ*_2_ (deg)	*λ*_2_	*l* (mm)	RMS (mm)
1	0.00	0.028	85.76	0.061	157.71	0.5411
2	0.25	0.183	70.00	0.223	183.14	0.4068
3	0.51	0.191	87.29	0.744	184.41	0.4228
4	0.76	0.728	73.90	0.752	185.25	0.3867
5	1.02	0.777	83.22	0.484	161.10	0.4108
6	1.27	0.199	89.66	0.109	156.44	0.5271
7	1.53	0.557	87.80	0.459	189.07	0.5251
8	1.78	0.085	71.19	0.427	168.73	0.5812
9	2.03	0.467	84.24	0.305	161.95	0.5672
10	2.29	0.264	75.08	0.321	155.17	0.4128
11	2.54	0.232	80.51	0.378	147.97	0.3603
12	2.80	0.288	81.53	0.834	177.63	0.4529
⋮	⋮	⋮	⋮	⋮	⋮	⋮
117	29.49	0.646	88.64	0.443	163.64	0.3627
118	29.75	0.817	84.75	0.516	168.31	0.3667
119	30.00	0.891	76.10	0.785	189.49	0.5792

**Table 3:** Basic parameters for numerical simulations.

Parameter	Value
Flow rate *Q*	2000 (m^3^/h)
Rotational speed	1400 r/min
Number of blades	6
Inlet operating pressure	1 (atm)

**Table 4:** The parameters of the Kriging model.

Parameter	Value
**β**	[2.688*e* − 5, −0.142, −0.062, −0.036, −0.093, 0.107]^T^
*σ* ^2^	0.00453
***θ***	[8.406, 14.718, 18.851, 21.512, 21.401]

**Table 5:** The parameter settings of MIGA.

Parameters	Value
Size of subpopulation	100
Number of islands	10
Number of generations	10
Gene size	32
Rate of crossover	1.0
Rate of mutation	0.01
Rate of migration	0.5
Interval of migration	5
Number of runs for the problem	30

**Table 6:** The result of optimization.

*φ*_1_ (deg)	*λ*_1_	*φ*_2_ (deg)	*λ*_2_	*l* (mm)	RMS (mm)	Average time (s)
26.37	0.938	83.31	0.934	156.89	0.3341	1836

**Table 7:** Results of Kriging, FSI simulation, and experiment.

	Kriging	FSI	Experiment
RMS (mm)	0.3341	0.3296	0.3447
